# Sex influences murine T cell responses to vaccination with BCG or BCG∆BCG1419 grown as biofilms

**DOI:** 10.1590/0074-02760250015

**Published:** 2025-12-12

**Authors:** Mario Alberto Flores-Valdez, Cristian Alfredo Segura-Cerda, César Pedroza-Roldán, Jorge Gómez-Haro, Dulce Mata-Espinosa, María Guadalupe Jorge-Espinoza

**Affiliations:** 1 Centro de Investigación y Asistencia en Tecnología y Diseño del Estado de Jalisco, Biotecnología Médica y Farmacéutica, Guadalajara, México; 2 Secretaría de Ciencia, Humanidades, Tecnología e Innovación, Ciudad de México, México; 3 Universidad de Guadalajara, Centro Universitario de Ciencias Biológicas y Agropecuarias, Departamento de Medicina Veterinaria, Zapopan, México; 4 Instituto Nacional de Ciencias Médicas y Nutrición Salvador Zubirán, Ciudad de México, México

**Keywords:** BCG, tuberculosis, biofilms, sex differences, immune response

## Abstract

**BACKGROUND:**

It is known that host sex can influence the immune response to administration of *Mycobacterium bovis* Bacillus Calmette-Guérin (BCG). However, the effect of BCG or BCG-derived vaccines cultured as biofilms on development of T cell responses in both sexes remains unclear.

**OBJECTIVES:**

To compare the influence of sex and vaccine strain (BCG Pasteur vs. BCGΔBCG1419c) on *ex vivo* T cell responses against mycobacterial purified protein derivative (PPD) stimulation in lung and spleen cells of mice vaccinated with bacteria grown as biofilms.

**METHODS:**

Male and female BALB/c mice were subcutaneously vaccinated with disaggregated, biofilm-derived BCG Pasteur or BCGΔBCG1419c. Sixty days later, lung and spleen cells were collected and stimulated *ex vivo* with PPD. Flow cytometry was used to quantify frequencies of mono- and bi-functional CD4⁺ and CD8a⁺ T cells expressing interferon gamma (IFN-γ) and tumour necrosis factor alpha (TNF-α) or interleukin-2 (IL-2), as well as frequencies of tissue-resident memory CD4⁺ T cells.

**FINDINGS:**

Sex influenced T cell responses in both organs. Lungs of female mice vaccinated with BCGΔBCG1419c showed reduced frequencies of CD8a⁺ IFN-γ⁺, and reduced frequency of CD4⁺ IFN-γ⁺ in spleen, compared with males. On the other hand, female mice vaccinated with BCG produced higher IL-2+ and IL-2+TNF-α+ T cells in spleen than paired males. Vaccine strain alone had limited effects, but sex-strain interactions shaped distinct immune profiles.

**MAIN CONCLUSIONS:**

Sex modulates the immunogenicity of BCG-based vaccines grown as biofilms. Our results underscore the importance of considering host sex and vaccine preparation in tuberculosis preclinical research.

Tuberculosis (TB) is a predominantly respiratory infectious disease with an estimated 10.8 million cases and around 1.25 million associated deaths in 2023.^1^ Although *Mycobacterium bovis* Bacille Calmette-Guèrin (BCG) remains the only licensed vaccine against TB, its efficacy, especially against adult pulmonary TB, is highly variable, ranging from 0% to 80% depending on geographic region and strain used.^1^ ^,^ ^2^

Among the challenges in developing new and efficacious vaccines against TB, two emerging factors have gained increasing attention in recent years. The first challenge is the growing body of evidence suggesting that sex influences both the course of TB infection and the protective efficacy of BCG-based vaccines. Epidemiological data consistently report a sex bias in TB prevalence, with men being nearly twice as likely as women to develop active TB.^1^ This disparity is not limited to human populations; it has also been confirmed in murine models.^3^ ^,^ ^4^ For instance, studies using C57BL/6 mice have shown that male mice experience more rapid weight loss, higher pulmonary bacterial burdens, and lower survival rates compared to females following *M. tuberculosis* infection.^5^ These sex differences in TB progression have been partially attributed to the immunomodulatory effects of sex hormones, particularly testosterone. In BALB/c mice, a relatively resistant strain, experimental evidence demonstrated that chemical castration in males reduced disease progression, supporting a testosterone-dependent mechanism underlying male susceptibility.^4^

The impact of sex on the efficacy of BCG vaccination was further investigated in a 2021 study using 129S2 mice, a highly TB-susceptible strain. This work showed that while BCG vaccination conferred protection against aerosol challenge with a low dose of *M. tuberculosis* H37Rv in both sexes, female mice exhibited lower bacterial loads and less weight loss during infection. Immunological analysis revealed that increased frequencies of CD8a⁺ T cells, CD8a⁺ central memory T cells, and CD4⁺ effector T cells correlated with improved protection in females. However, this study did not address whether sex influences the immunogenicity of BCG in unchallenged, vaccinated animals.^6^ This evidence highlights a critical gap in our understanding of the immune response to mycobacteria: while the role of sex in TB disease progression is increasingly recognised, the influence of sex on vaccine-induced immune responses prior to infection remains poorly understood. Addressing this question is essential for optimising TB vaccine strategies and ensuring they are effective across sexes.

A second emerging challenge in the development of new BCG-derived vaccines is the growing evidence that production methods can significantly influence vaccine immunogenicity and efficacy.^2^ The culture conditions under which BCG is grown have been shown to alter the host immune response. For example, studies have demonstrated that preparation techniques such as sonication and filtration can affect bacterial integrity and antigen presentation, resulting in variability in experimental outcomes.^7^

Additional *in vitro* evidence supports this notion. In one study, THP-1 cells differentiated into macrophages were infected with *M. tuberculosis* grown either in planktonic form or as biofilms. The biofilm-derived bacteria, produced using a Rotary Cell Culture System, induced significantly higher levels of tumour necrosis factor alpha (TNF-α) compared to their planktonic counterparts, suggesting that bacterial phenotype strongly influences macrophage activation.^8^

Importantly, *in vivo* studies have corroborated the impact of BCG growth conditions on host response to mycobacteria. Fitzpatrick et al.^9^ reported that guinea pigs vaccinated with biofilm (pellicle)-grown BCG exhibited better protection against *M. tuberculosis* challenge than those vaccinated with conventionally cultured, planktonic BCG. This study provides compelling evidence that *in vitro* growth conditions, specifically, planktonic versus biofilm-derived preparations, can directly impact vaccine efficacy in a relevant animal model.

While these findings highlight a critical variable in BCG vaccine production, the impact of such differences on human immunogenicity and protection remains to be formally evaluated. These gaps in knowledge are particularly relevant as both sex and production methods are often overlooked in preclinical vaccine evaluation.

Given these challenges, we aimed to investigate the combined influence of sex and BCG culture conditions on the immunogenicity of BCG, specifically on T CD4^+^ and T CD8a^+^ populations. As we have previously characterised some aspects of the T cell response to BCG and the BCGΔBCG1419c strain cultured as planktonic bacteria^10^ ^,^ ^11^ we here characterised the T cell response against biofilm cultured bacteria.

The BCGΔBCG1419c is a vaccine candidate lacking the *BCG1419c* gene,^12^ which form robust *in vitro* biofilms and has demonstrated enhanced immunogenicity and reduced pulmonary pathology in various animal models. Previous studies from our group have shown that BCGΔBCG1419c elicits improved immune responses and greater protective efficacy compared to wild-type BCG when both are cultured under conventional planktonic conditions.^11^ ^,^ ^13^ ^,^ ^14^

Moreover, proteomic analyses have revealed that several immunogenic proteins are differentially expressed in BCGΔBCG1419c compared to BCG, and that these differences persist regardless of whether the bacteria are grown in 7H9-OADC-Tween (planktonic) or Sauton medium (biofilm conditions).^13^ ^,^ ^15^

While several aspects of BCGΔBCG1419c immunogenicity have been characterised, the role of sex and bacterial growth mode in shaping vaccine-induced T cell responses have not been explored. In this study, we aimed to determine how sex and the use of a recombinant BCG strain (BCGΔBCG1419c) or its parental strain BCG Pasteur ATCC 35734, both grown under biofilm conditions, influence the magnitude of T cell responses in BALB/c mice. We hypothesised that sex-vaccine interactions modulate T cell immunity in a strain-dependent manner, which could have implications for TB vaccine development.

Our data reveal that sex significantly shapes T cell responses to BCG vaccination, with distinct patterns depending on the vaccine strain. These findings highlight the relevance of sex-vaccine interactions and support the inclusion of sex as a critical variable in TB vaccine development even from the preclinical stage.

## MATERIALS AND METHODS

*Strains and culture conditions* - BCG Pasteur ATCC 35734 was used as parental strain and the antibiotic-less version of BCGΔBCG1419c was used for comparative purposes. Both strains were cultured in Sauton medium (containing per litre: asparagine 4.0 g, citric acid 2.0 g, K_2_HPO_4_ 0.5 g, MgSO_4_ 0.5 g, ferric ammonium citrate 0.05 g, glycerol 60 mL, 1% ZnSO_4_, 1 mL, pH 7.0-7.4) with 0.05% Tween 80 at 37ºC, 100 rpm, up to an OD_600 nm_ ≈ 0.8 (mid-late log phase). When both strains reached this OD_600 nm_ value, we harvested them by centrifugation at 5000 rpm, 5 min, washed them with Sauton with no detergent, and started cultures in Sauton with no detergent, within vented cap, 75 cm^2^ tissue cultures flasks, at an OD_600 nm_ ≈ 0.03 and incubated them at 37ºC, 5% CO_2_, for two weeks. After this, surface pellicles of each strain were harvested, transferred into sterile 50 mL tubes, centrifuged 5 min at 5000 rpm, room temperature, with the supernatant being discarded and cells were washed once with sterile 1X phosphate-buffered saline (PBS). After this, an aliquot of 15 mg from each strain was placed into screw-capped, 2 mL tubes containing 5 mm sterile steel beads. One mL of sterile 1X PBS was added to each tube and cells were mixed by vortex. This step was repeated two-three more times until no clumps were visually detected. Then, cells were further disaggregated by using insulin syringes and checked by Kinyoun staining to confirm cells were visually intact (that is, not damaged, broken, or with big debris arising from this procedure). OD_600 nm_ was read, and serial dilutions were prepared for vaccination and colony-forming units (CFU) enumeration by plating onto 7H10 OADC (Oleic acid, Albumin, Dextrose, Catalase) agar plates, incubated at 37ºC, 5% CO_2_ for three weeks. All these procedures were performed within biosafety cabinets.

*Vaccination and evaluation of ex vivo T cell responses* - Pathogen-free, six-eight weeks old, male and female BALB/c mice were obtained from Bioterio Morelos (Mexico). Groups of mice (n = 5) were maintained in ventilated cages (n = 5 mice per cage) and were randomly allocated to receive either BCG or BCGΔBCG1419c, applied subcutaneously at the base of the tail with ~10^5^ CFU suspended in 50 μL of saline solution. We used this vaccine dose to reflect the ones used in a human dose (5 × 10^5^ CFU).

Calculation of sample size was performed following the Charan-Kantharia equation, calculated for the main responses to evaluate (flow cytometry) during the study considering a confidence interval of 95% and a statistical power of 80%.

**Fig. 1:**
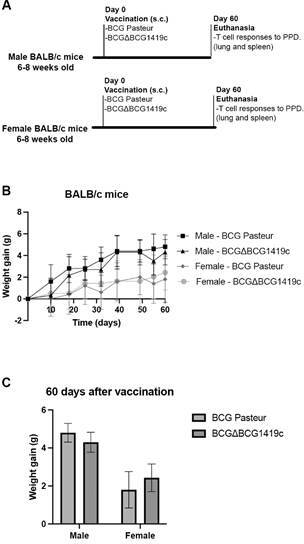
(A) Experimental design. Specific pathogen-free BALB/c mice (male or female, six-eight weeks old) were subcutaneously vaccinated with either Bacillus Calmette-Guérin (BCG) Pasteur or BCGΔBCG1419c (n = 5 mice per group). Sixty days post-vaccination, T cell responses to purified protein derivative (PPD) were assessed in the lungs and spleen. (B) Variation of body weight gain over the experiment. Body weight was recorded throughout the experiment, and weight gain was calculated relative to the pre-vaccination weight. Data points represent the mean weight gain per group, with error bars indicating the standard deviation. (C) Weight gain at 60 days post-vaccination in male and female mice, vaccinated with BCG or BCG∆BCG1419c. Bars indicate group means, and error bars represent standard deviation. Statistical comparisons of weight gain across groups were performed using two-way analysis of variance (ANOVA) followed by Tukey’s post hoc test, with significance defined as p < 0.05.

Sixty days after vaccination, mice were humanely euthanised by cervical dislocation, without anaesthesia by an experimented researcher (CPR), who confirmed separation of the spinal cord from the skull before obtaining lung and spleen samples (Fig. 1A). Evaluation of lung and spleen immunogenicity against mycobacteria was performed in each mouse.

*Evaluation of T cell response in lungs and spleen* - To evaluate the immunogenicity of biofilm-cultured BCG and BCGΔBCG1419c strains, we analysed T cell responses in the lungs and spleens of male and female BALB/c mice. After euthanasia, lungs and spleens were aseptically harvested and processed separately under sterile conditions. Lungs were enzymatically digested using collagenase type II (Gibco, 17101015) at 1 μg/mL, followed by mechanical disaggregation through sequential passage with 18G and 21G needles. Spleens were perfused with 1.5 mL of Roswell Park Memorial Institute medium (RPMI) 1640 (Sigma-Aldrich, R8758) and centrifuged at 2000 rpm for 10 min at room temperature to collect mononuclear cells. For both organs, red blood cells were lysed using ammonium chloride lysis buffer (Biolegend, 420301), and the resulting cell suspensions were filtered through 70 μm nylon strainers (Corning, 352350). Cells were resuspended in complete RPMI medium supplemented with 10% foetal bovine serum (FBS, Thermo Fisher, 10437028) and antibiotic-antimycotic solution (Sigma-Aldrich, A5955). Cell viability was assessed by trypan blue exclusion using an automated cell counter (Luna II, Logos Biosystems).

For *ex vivo* stimulation, lung and spleen cells were seeded in round-bottom 96-well plates at densities of 1 × 10⁶ and 0.5 × 10⁶ cells per well, respectively. Stimulation was performed in complete RPMI medium with or without purified protein derivative (PPD, PRONABIVE, B-0653-035) at a final concentration of 5 μg/μL. For lung samples, anti-CD28 (Cytek, 70-0281) was added at 1 μg/mL as a co-stimulatory signal. All cultures were incubated at 37ºC with 5% CO₂ for 24 h. Six hours before harvest, 5 μg/mL brefeldin A (Biolegend, 420601) were added to block cytokine secretion and allow intracellular accumulation.

Surface and intracellular staining were performed on both lung and spleen samples following organ-appropriate protocols. For lung cells, Fc receptors were blocked with purified anti-mouse CD16/CD32 antibody (Cytek, 70-0161) at 0.5 μg/sample for 20 min at 4ºC. Cells were then incubated for 30 min at 4ºC in the dark with fluorochrome-conjugated monoclonal antibodies diluted in staining buffer: anti-CD3-FITC (Fluorescein isothiocyanate*,* Biolegend, 100204), anti-CD4-PerCP/Cy5.5 (Peridinin-chlorophyll-protein complex/Cyanine 5.5 tandem dye*,* Cytek, 65-0042), anti-CD8a-PerCP/Cy5.5 (Peridinin-chlorophyll-protein complex/Cyanine 5.5 tandem dye, Cytek, 65-0081), anti-CD103-PE (Phycoerythrin , Biolegend, 121405), and anti-CD69-APC (Allophycocyanin, Cytek, 20-0691). After staining, cells were washed and fixed/permeabilised using the eBioscience™ Intracellular Fixation & Permeabilisation Buffer Set (Invitrogen, 88-8824-00), followed by intracellular staining with anti-IFN-γ-APC (Interferon gamma, Biolegend, 505810) and anti-TNF-α-PE (Biolegend, 506104), both used at 0.1 μg per sample.

For spleen cells, surface staining was performed with anti-CD4-PE (Biolegend, 100408) and anti-CD8a-FITC (Biolegend, 100706), incubated at 4ºC for 30 min in the dark. Cells were then fixed and permeabilised using Cytofix/Cytoperm™ Fixation/Permeabilisation Kit (BD Biosciences, 554722), and intracellular staining was performed with anti-IFN-γ-PerCP/Cy5.5 (Biolegend, 505826) and anti-TNF-α-APC (Biolegend, 506310), interleukin-2 (IL-2)-PerCp-Cy5.5 (Biolegend, 503822) or anti-Perforin-APC (Biolegend 154304), also at 0.1 μg per sample. Stained cells were suspended in 1× PBS with 2% bovine serum albumin (BSA) (Sigma-Aldrich, A7906) and stored at 4ºC, protected from light, until flow cytometry acquisition.

For all samples, appropriate controls were included, including unstained cells, single-colour stained controls, and fluorescence-minus-one (FMO) controls for each fluorochrome. Acquisition was performed on a BD Accuri™ C6 Plus flow cytometer (BD Biosciences), with a minimum of 100,000 events recorded per sample. The cytometer is configured with four blue lasers and one red laser, equipped with the following detectors: 533/30 (for FITC-conjugated antibodies), 670 LP (for PerCp-Cy5.5-conjugated antibodies), 675/24 (for APC-conjugated antibodies), and 585/40 (for PE-conjugated antibodies). In the gating strategy, the molecule, fluorochrome, and detector are indicated; however, in the text only the molecule is mentioned to improve readability. Data analysis was conducted using BD C6 Plus Software and FlowJo v10 (BD), applying consistent gating strategies as shown in Supplementary data (Fig. 1). For cell analysis, we identified frequencies of bifunctional CD4⁺ helper T cells (CD3⁺ CD4⁺ IFN-γ⁺ TNF-α⁺), bifunctional CD8a⁺ cytotoxic T cells (CD3⁺ CD8a⁺ IFN-γ⁺ TNF-α⁺), and tissue-resident memory T cells (CD3⁺ CD4⁺ CD69⁺ CD103⁺) in lung samples. In spleen samples, we assessed the same bifunctional CD4⁺ and CD8a⁺ subsets plus IL-2^+^ cells and Perforin^+^ populations based on intracellular cytokine positivity.

*Statistical analysis* - Data are presented with means plus the standard deviation, and then the means compared with a two-way analysis of variance (ANOVA), including sex and vaccine as factors. A limit of α = 0.05 was established to discriminate differences between groups. Analysis was performed in the software GraphPad Prism v10 for Mac OS X.

*Ethics approval* - The Internal Committee for the Care and Use of Laboratory Animals at CIATEJ approved the experiments with project number 2023-010A. The animal research adheres to the ARRIVE guidelines (https://arriveguidelines.org/arrive-guidelines). All experiments complied with the Mexican guidelines regarding ethical and safe handling of experimental animals and final disposal of materials such as: NOM-07- SEMARNAT-SSA1-2002, NOM-033-ZOO-1995, and NOM-062- ZOO-1999.

## RESULTS

*Vaccination with biofilm-derived BCG or BCG∆BCG1419c does not affect weight gain in male or female BALB/c mice* - To gain insight into the effect of vaccination with biofilm-derived BCG or BCG∆BCG1419c, we vaccinated male or female BALB/c mice and assessed T cell responses at day 60 post-vaccination (Fig. 1A), as well as weight gain over time. Consistent with our previous findings using planktonic bacteria to vaccinate female BALB/c and C57BL/6 mice,^2^ no significant effect on body weight gain was observed over time when BCG or BCG∆BCG1419c-biofilm derived bacteria were used to vaccinate BALB/c mice (this work). As expected, female mice consistently gained less body weight than males throughout the experiment (Fig. 1B); however, vaccination had no impact on weight gain in either sex during the 60-day observation period (Fig. 1C).

**Fig. 2:**
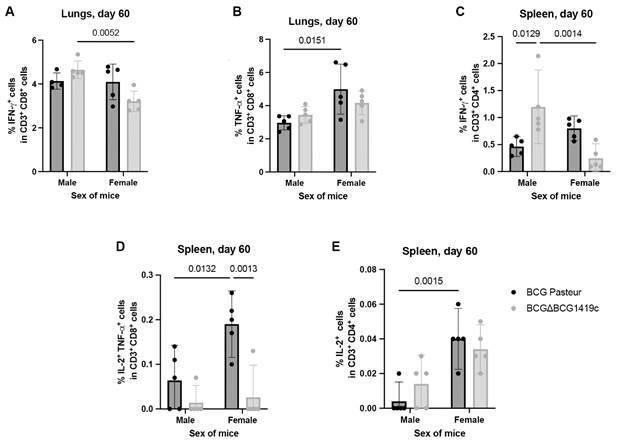
T cell response to purified protein derivative (PPD) in male and female mice vaccinated with Bacillus Calmette-Guérin (BCG) or BCGΔBCG1419c cultured as biofilms. BALB/c mice (male and female) were subcutaneously vaccinated with biofilm-derived BCG Pasteur or BCGΔBCG1419c. Sixty days post-vaccination, lung and spleen leukocytes were stimulated *ex vivo* with PPD and analysed by flow cytometry. (A) Frequency of CD3⁺CD8a⁺ interferon gamma (IFN-γ)⁺ T cells in CD3⁺CD8a⁺ cells. (B) Frequency of CD3⁺CD8a⁺ tumour necrosis factor alpha (TNF-α)⁺ T cells in CD3⁺CD8a⁺ cells. (C) Frequency CD3⁺CD4⁺IFN-γ⁺ T cells in CD3⁺CD4⁺ cells. (D) Frequency of CD3⁺CD8a⁺ interleukin-2 (IL-2)⁺ TNF-α⁺ T cells in CD3⁺CD8a⁺ cells. (E) Frequency of CD3⁺CD4⁺ IL-2⁺ T cells in CD3⁺CD4⁺ cells. Each dot represents an individual animal; horizontal lines indicate group means and vertical lines standard deviation. Statistical analyses were performed using two-way analysis of variance (ANOVA) followed by Tukey’s post hoc test. Significant p-values are indicated in the panels.

*Sex modifies T cell response ex vivo to PPD in lungs and spleen of mice vaccinated with biofilm-derived BCG or BCG∆BCG1419c* - To clarify the influence of sex on T cell responses, we vaccinated male and female mice and evaluated *ex vivo* recall responses to PPD in lung-derived lymphocytes. No significant sex-related differences were observed in the frequencies of CD3⁺ CD4⁺ IFN-γ⁺, CD3⁺ CD4⁺ TNF-α⁺, CD3⁺ CD4⁺ IFN-γ⁺ TNF-α⁺, or memory T cells (CD3⁺ CD4⁺ CD103⁺ CD69⁺) in the lungs [Supplementary data (Fig. 2)]. However, sex significantly influenced subpopulations of CD8a⁺ T cell responses.

Specifically, in mice vaccinated with BCGΔBCG1419c, *ex vivo* stimulation with PPD induced significantly lower frequencies of CD3⁺ CD8a⁺ IFN-γ⁺ lymphocytes in females compared to males receiving the same vaccine (p = 0.0052, Fig. 2A). Conversely, among mice vaccinated with BCG Pasteur, PPD stimulation elicited higher frequencies of CD3⁺ CD8a⁺ TNF-α⁺ lymphocytes in females than in males (p = 0.0151, Fig. 2B). The two-way ANOVA analysis revealed that the vaccine strain had no significant effect on the induction of CD3⁺ CD8a⁺ IFN-γ⁺ or CD3⁺ CD8a⁺ TNF-α⁺ lymphocytes (p = 0.4748 and p = 0.6628, respectively), whereas sex had a significant effect on both populations (p = 0.0106 and p = 0.0035, respectively). Furthermore, there was a significant interaction between sex and vaccine strain for CD3⁺ CD8a⁺ IFN-γ⁺ lymphocytes (p = 0.0154) while on CD3⁺ CD8a⁺ TNF-α⁺ lymphocytes not (p = 0.1227), as shown in Supplementary data (Tables I-II). These findings suggest that sex-specific host-vaccine interactions modulate CD8a⁺ T cell responses in lung following BCG vaccination.

In the spleen, no significant differences attributable to sex or vaccination were observed in the frequencies of CD3⁺ CD8a⁺ IFN-γ⁺, CD3⁺ CD8a⁺ TNF-α⁺, CD3⁺ CD8a⁺ IFN-γ⁺ TNF-α⁺, CD3⁺ CD4⁺ IFN-γ⁺ TNF-α⁺, CD3⁺ CD8a⁺ IL-2⁺, or CD3⁺ CD8a⁺ Perforin⁺ lymphocytes following PPD stimulation. However, sex significantly influenced other T cell subsets.

Female mice vaccinated with BCGΔBCG1419c exhibited significantly lower frequencies of CD3⁺ CD4⁺ IFN-γ⁺ lymphocytes compared to male counterparts receiving the same vaccine (p = 0.0014, Fig. 2C). In contrast, female mice vaccinated with BCG Pasteur showed higher frequencies of CD3⁺ CD8a⁺ IL-2⁺ TNF-α⁺ lymphocytes than similarly vaccinated males (p = 0.0132, Fig. 2D). Additionally, the frequency of CD3⁺ CD4⁺ IL-2⁺ lymphocytes was significantly higher in females than in males, irrespective of the vaccine strain (p = 0.0015, Fig. 2E).

As observed in the lungs, the vaccine strain did not significantly affect the frequencies of CD3⁺ CD4⁺ IFN-γ⁺ (p = 0.5359) or CD3⁺ CD4⁺ IL-2⁺ (p = 0.7175) lymphocytes. However, vaccine type did significantly influence the induction of CD3⁺ CD8a⁺ IL-2⁺ TNF-α⁺ cells (p = 0.0005). Two-way ANOVA also indicated significant interactions between sex and vaccine strain in the modulation of CD3⁺ CD4⁺ IFN-γ⁺ [p = 0.0003, Supplementary data (Table III)] and CD3⁺ CD8a⁺ IL-2⁺ TNF-α⁺ [p = 0.0334, Supplementary data (Table IV)] lymphocyte frequencies, while no interaction effect was found for CD3⁺ CD8a⁺ IL-2⁺ lymphocytes alone [p = 0.1602, Supplementary data (Table V)]. These findings further support the notion that the host immune response to BCG vaccination in the spleen is shaped by both sex and vaccine strain, and that sex-vaccine interactions contribute to distinct immunological outcomes in terms of T cell responses.

## DISCUSSION

Previous studies from our group using planktonic cultures of BCGΔBCG1419c demonstrated variable immunogenicity depending on the strain version and experimental conditions. The first-generation, hygromycin-resistant BCGΔBCG1419c vaccine showed increased frequencies of CD4⁺ and CD8a⁺ IFN-γ-producing T cells in spleens of vaccinated female BALB/c mice than BCG-vaccinated ones,^16^ ^)^ whereas the second-generation, antibiotic-free version of BCGΔBCG1419c induced lower frequencies of these populations in male BALB/c mice than BCG-vaccinated group.^17^ Given that both studies used planktonic bacteria grown in 7H9-OADC-Tween medium, and that no major genomic differences were identified with BCG Pasteur ATCC 35734, the strain where BCGΔBCG1419c comes from,^18^ we hypothesised that sex- and strain-dependent immune responses could explain these discrepancies.

In contrast to these earlier evaluations, the present study was designed to assess immune responses induced by BCG Pasteur and BCGΔBCG1419c (second-generation, antibiotic-less, Pasteur ATCC 35734-derived) when grown as biofilms, a condition that better reflects manufacturing protocols for human-use BCG vaccines, a process that remains mostly unchanged since the 1920’s and that has driven several shortcomings including availability issues in recent years, but that given the low BCG vaccine prices have reduced interest in investing in new production protocols.^19^ Therefore, to better reflect current BCG vaccine manufacturing processes, we decided to grow BCG and BCG∆BCG1419c as biofilms and both strains were mechanically disaggregated before vaccination to simulate industrial preparations. Under these conditions, we observed sex-dependent differences in T cell responses. In lungs, male mice vaccinated with BCGΔBCG1419c had higher frequencies of CD8a⁺ IFN-γ⁺ and CD4⁺ resident memory T cells, while females vaccinated with BCG Pasteur showed increased CD8a⁺ TNF-α⁺ responses. In spleens, BCGΔBCG1419c elicited greater CD4⁺ IFN-γ⁺ and TNF-α⁺ responses in males, whereas BCG Pasteur enhanced CD4⁺ and CD8a⁺ TNF-α⁺ responses in females.

Interestingly, although BCG Pasteur appeared to induce fewer overall changes in the observed T cell populations, these changes seemed more frequent in females, while BCGΔBCG1419c tended to elicit broader changes in males, which might be in agreement with its recently reported improved efficacy compared with BCG Pasteur to protect male C57BL/6 mice against challenge with the hypervirulent Mtb HN878 strain.^20^ This apparent dual pattern may suggest an interplay between vaccine strain and host sex, raising the possibility that immunogenicity could, at least at the level of some T cell subsets, display sex-related differences, a factor rarely explored in preclinical TB vaccine studies. Taken together, these findings support the notion that immunogenicity and host responses may not only be vaccine-specific but could also be influenced by sex.

Additionally, when comparing responses between strains, BCG Pasteur induced higher CD4⁺ TNF-α⁺ frequencies in lungs of male mice, while in females it was associated with greater CD4⁺ and CD8a⁺ TNF-α⁺ responses in spleens. Conversely, BCGΔBCG1419c increased CD4⁺ IFN-γ⁺ cells in spleens of male mice, further supporting the idea that strain-specific immune signatures are shaped by host sex.

A limitation of this study is the absence of a direct comparison with vaccines grown under conventional planktonic conditions. However, our objective here was to determine whether vaccines prepared under biofilm conditions, more reflective of industrial production, could still reveal differential patterns of immunogenicity based on sex and strain. The restricted panel of T cell populations examined also represents a conservative starting point, with future work needed to evaluate additional immune parameters, including innate responses, histopathological correlates, and protective efficacy.

In conclusion, our findings demonstrate that biofilm-derived BCG and BCGΔBCG1419c induce distinct, sex-dependent T cell responses in both lung and spleen of BALB/c mice. These observations not only advance our understanding of host-vaccine interactions but also emphasise the importance of incorporating sex and manufacturing-relevant bacterial phenotypes into the design and evaluation of next-generation TB vaccines.

## Supplementary Material

Supplementary PDF file

## Data Availability

The contents underlying the research text are included in the manuscript. All data generated or analysed during this study are included in this article and its supplementary information files.
