# Supplementary material for: Sex influences murine T cell responses to vaccination with BCG or BCG∆BCG1419 grown as biofilms
Source: Mem Inst Oswaldo Cruz. 2025 Dec 12;120:e250015. doi: 10.1590/0074-02760250015 (PMC12711205; doi:10.1590/0074-02760250015)
Supplement: Supplementary PDF file [file 1678-8060-mioc-120-e250015-s.pdf]

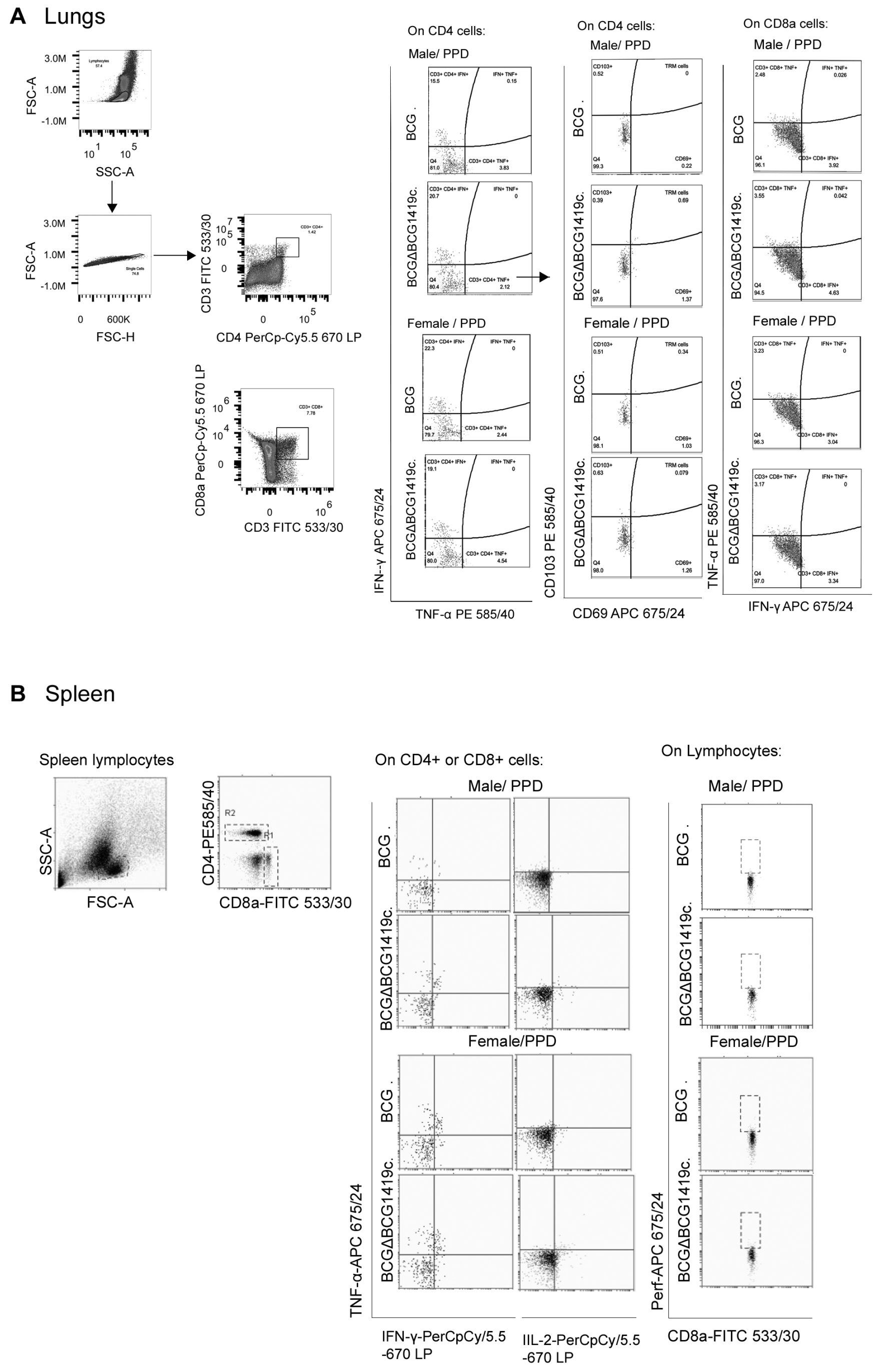

Fig. 1: representative of gating strategy followed to determine cell frequencies in organs of vaccinated-male and female mice. (A) For lungs, we first identified the region of the lymphocytes on a view of SSA and FCS-A. On this, single cells were chosen on a FSC-A vs FCS-H view. On lung single cells, a double-gating of CD4-PerCp-Cy5.5 (limit at 10^3) and CD3-FITC (limit at 1 x 10^3) was done to identify $CD3^+CD4^+$ T cells. (A, left panel) Then, cells were gated on of IFN-γ-APC (limit at 1 x1 0^3) and TNF-α-PE (limit at 10^3) to identify single and double positive IFN- γ^+ TNF-α^+ T cells. Panels for a mouse representative for each condition is shown. (A, centre panel) In another panel, on $CD3^+CD4^+$ T cells, cells were gated on of CD69-APC (limit at 1 x 10^3) and CD103-PE (limit at 10^3) to identify resident, double positive, T cells. Panels for a representative mouse for each condition is shown. (A, right panel) On lung single cells, a double-gating of CD8a-PerCp-Cy5.5 (limit at 10^3) and CD3-FITC (limit at 1 x 10^3) was done to identify $CD3^+CD8a^+$ T cells. Then, cells were gated on of IFN-γ-APC (limit at 1 x 10^3) and TNF- α-PE (limit at 10^3) to identify single and double positive IFN- γ^+TNF-α^+ T cells. Panels for a mice representative for each condition is shown. (B) Spleen cells were first identified on the region of the lymphocytes on a view of SSA and FCS-A. On lymphocytes, a double-gating of CD4-PE (limit at 10^3) and CD8a-FITC (limit at 1 x 10^3) was done to identify $CD4^+$ or $CD8a^+$ T cells. (B, left panel) Then, cells were gated on of IFN-γ-PerCp-Cy5.5 (limit at 1 x 10^3) and TNF-α-APC (limit at 10^3) to identify single and double positive IFN- γ^+TNF-α^+ T cells. Panels for a representative mouse for each condition is shown. (B, centre panel) Alternatively, $CD4^+$ or $CD8a^+$ T cells were gated on of IL-2-PerCp-Cy5.5 (limit at 1 x 10^3) and TNF-α-APC (limit at 10^3) to identify single and double positive IL-2^+ TNF-α^+ T cells. Panels for a representative mouse for each condition is shown. (B, right panel) Separately, lymphocytes were gated on CD8a-FITC (limit at 1 x 10^3) and Perforin-APC (limit at 10^3) to identify $CD8a^+$ IL-2^+ T cells. Panels for a representative mouse for each condition is shown.

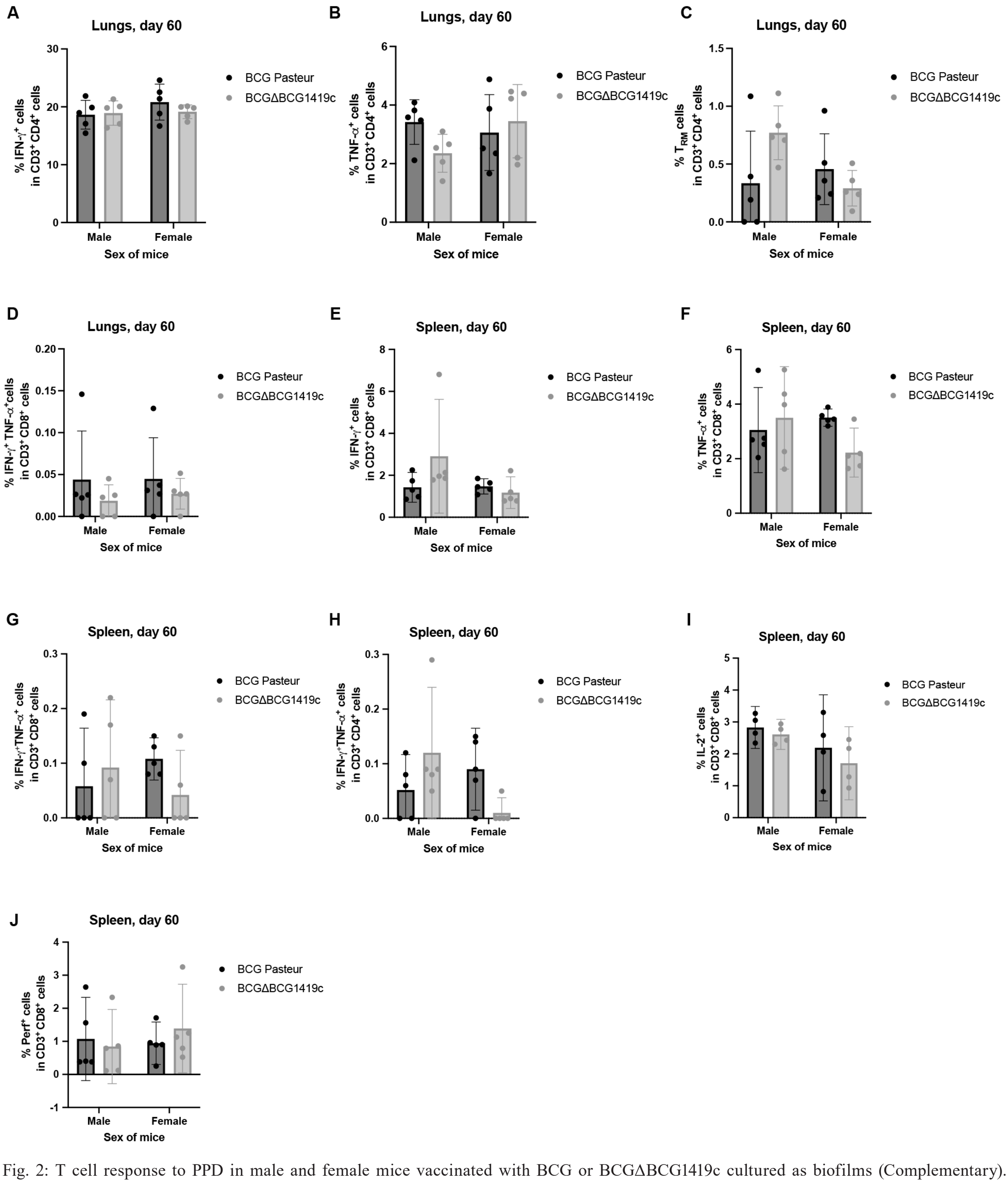

Fig. 2: T cell response to PPD in male and female mice vaccinated with BCG or BCGΔBCG1419c cultured as biofilms (Complementary). BALB/c mice (male or female) were subcutaneously vaccinated with biofilm-cultured BCG Pasteur or BCGΔBCG1419c. Sixty days post-vaccination, lung and spleen leukocytes were stimulated *ex vivo* with PPD and analysed by flow cytometry. (A) Frequencies of $CD3^+CD4^+$ IFN-γ^+ T cells in $CD3^+CD4^+$ cells of lungs. (B) Frequencies of $CD3^+CD4^+$ TNF-α^+ T cells in $CD3^+CD4^+$ cells in lungs. (C) Frequencies of resident T cell lymphocytes in $CD3^+$ $CD4^+$ cells in lungs. (D) Frequencies of IFN-γ^+ TNF-α^+ cells in $CD3^+CD8a^+$ cells in lungs. (E) Frequencies of IFN-γ^+ cells in $CD3^+CD8a^+$ cells in spleen. (F) Frequencies of TNF-α^+ cells in $CD3^+CD8a^+$ cells in spleen. (G) Frequencies of IFN-γ^+ TNF-α^+ cells in $CD3^+CD8a^+$ cells in spleen. (H) Frequencies of IFN-γ^+ TNF-α^+cells in $CD3^+CD4^+$ cells in spleen. (I) Frequencies of IL-2^+ cells in $CD3^+CD8a^+$ cells in spleen. (J) Frequencies of Perforin $(Perf)^+$ cells in $CD3^+CD8a^+$ cells in spleen. Each dot represents an individual mouse; horizontal lines indicate group means and vertical lines standard deviation. Statistical analyses were performed using two-way ANOVA followed by Tukey's post hoc test. Significant p-values are indicated in the panels.

TABLE I

Two-way analysis of variance (ANOVA) for the frequencies of $CD3^+$ $CD8a^+$ IFN-γ^+ lymphocytes in lungs of male and female mice vaccinated with *Mycobacterium bovis* Bacille Calmette-Guerin (BCG) or BCGΔBCG1419c

ANOVA Table	SS (Type III)	DF	MS	F (DFn, DFd)	p-value
Interaction sex-vaccine	2.321	1	2.321	F (1, 15) = 7.646	p = 0.0144
Sex (male or female)	2.586	1	2.586	F (1, 15) = 8.519	p = 0.0106
Vaccine (BCG or BCGΔBCG1419c)	0.1631	1	0.1631	F (1, 15) = 0.5374	p = 0.4748
Residual	4.554	15	0.3036		

SS: quadratic error sum; DF: degrees of freedom; MS: mean square; F: value of F distribution; p: probability of difference induced by chance.

TABLE II

Two-way analysis of variance (ANOVA) for the frequencies of $CD3^+CD8a^+$TNF-α^+ lymphocytes in lungs of male and female mice vaccinated with *Mycobacterium bovis* Bacille Calmette-Guerin (BCG) or BCGΔBCG1419c

ANOVA Table	SS	DF	MS	F (DFn, DFd)	p-value
Interaction sex-vaccine	2.128	1	2.128	F (1, 16) = 2.656	p = 0.1227
Sex (male or female)	9.405	1	9.405	F (1, 16) = 11.74	p = 0.0035
Vaccine (BCG or BCGΔBCG1419c)	0.1572	1	0.1572	F (1, 16) = 0.1962	p = 0.6638
Residual	12.82	16	0.8012		

SS: quadratic error sum; DF: degrees of freedom; MS: mean square; F: value of F distribution; p: probability of difference induced by chance.

TABLE III

Two-way analysis of variance (ANOVA) for the frequencies of $CD3^+$ $CD4^+$ IFN-γ^+ lymphocytes in spleen of male and female mice vaccinated with *Mycobacterium bovis* Bacille Calmette-Guerin (BCG) or BCGΔBCG1419c

ANOVA Table	SS	DF	MS	F (DFn, DFd)	p-value
Interaction sex-vaccine	2.074	1	2.074	F (1, 16) = 20.50	p = 0.0003
Sex (male or female)	0.4743	1	0.4743	F (1, 16) = 4.688	p = 0.0458
Vaccine (BCG or BCGΔBCG1419c)	0.04050	1	0.04050	F (1, 16) = 0.4003	p = 0.5359
Residual	1.619	16	0.1012		

SS: quadratic error sum; DF: degrees of freedom; MS: mean square; F: value of F distribution; p: probability of difference induced by chance.

TABLE IV

Two-way analysis of variance (ANOVA) for the frequencies of $CD3^+$ $CD8a^+$ $IL\text{-}2^+$ $TNF\text{-}\alpha^+$ lymphocytes in spleen of male and female mice vaccinated with *Mycobacterium bovis* Bacille Calmette-Guerin (BCG9 or BCGΔBCG1419c

ANOVA Table	SS	DF	MS	F (DFn, DFd)	p-value
Interaction	0.01625	1	0.01625	F (1, 16) = 5.420	p = 0.0334
Sex (male or female)	0.02381	1	0.02381	F (1, 16) = 7.942	p = 0.0124
Vaccine (BCG or BCGΔBCG1419c)	0.05725	1	0.05725	F (1, 16) = 19.10	p = 0.0005
Residual	0.04796	16	0.002998		

SS: quadratic error sum; DF: degrees of freedom; MS: mean square; F: value of F distribution; p: probability of difference induced by chance.

TABLE V

Two-way analysis of variance (ANOVA) for the frequencies of $CD3^+$ $CD8a^+$ $IL\text{-}2^+$ lymphocytes in spleen of male and female mice vaccinated with *Mycobacterium bovis* Bacille Calmette-Guerin (BCG) or BCGΔBCG1419c

ANOVA Table	SS	DF	MS	F (DFn, DFd)	p-value
Interaction	0.0003200	1	0.0003200	F (1, 16) = 2.169	p = 0.1602
Sex (male or female)	0.003920	1	0.003920	F (1, 16) = 26.58	p < 0.0001
Vaccine (BCG or BCGΔBCG1419c)	2.000e-005	1	2.000e-005	F (1, 16) = 0.1356	p = 0.7175
Residual	0.002360	16	0.0001475		

SS: quadratic error sum; DF: degrees of freedom; MS: mean square; F: value of F distribution; p: probability of difference induced by chance.
